# Factors associated with awareness and practice about foot care among patients admitted with diabetes mellitus: A cross sectional research from a medical college hospital of southern India

**DOI:** 10.3126/nje.v10i3.29213

**Published:** 2020-09-30

**Authors:** H Pavithra, Kibballi Madhukeshwar Akshaya, Abhay Subashrao Nirgude, AG Balakrishna

**Affiliations:** 1 Department of Community Medicine, Yenepoya Medical College, Yenepoya (Deemed to be University), Mangaluru, Karnataka, India, 575018; 2 Department of Community Medicine, Yenepoya Medical College, Yenepoya (Deemed to be University), Mangaluru, Karnataka, India, 575018; 3 Department of Community Medicine, Yenepoya Medical College, Yenepoya (Deemed to be University), Mangaluru, Karnataka, India, 575018; 4 Department of Community Medicine, Yenepoya Medical College, Yenepoya (Deemed to be University), Mangaluru, Karnataka, India, 575018

**Keywords:** Knowledge, Diabetic foot, Diabetes complications, Health education, Tertiary Care Center

## Abstract

**Background:**

Diabetes Mellitus (DM) causes micro and macro vascular complications. One of the complications of DM is diabetic foot that results in amputations and decreased quality of life. The aim of this study was to assess the awareness and practice about foot care and associated factors among admitted patients in a teaching hospital of coastal Karnataka, India.

**Material and Methods:**

A cross-sectional study was conducted in a medical college hospital after obtaining institutional ethics approval from 24th December 2016 to 21st January 2017. Adults with diabetes (N=317) admitted in the hospital were interviewed with a validated structured questionnaire for awareness and practice regarding foot care. The scores obtained were further graded into good and poor. Data was analyzed with SPSS version 22 for descriptive statistics. Bivariate logistic and linear regressions were used to determine the association between variables and awareness/practice scores.

**Results:**

Mean age of the participants was 56.98 (±10.54) years with males constituting the majority (63.4%). Good awareness and practice scores were observed among 69.1% and 41.6% participants, respectively. Good awareness scores were associated with male patients (p=0.027), currently not married (p=0.044), below poverty line socioeconomic status (p=0.014) and presence of foot ulcer (p=0.021). Good practice scores was associated with secondary schooling (p=0.003) and receiving insulin (p=0.045). Moderate correlation with coefficient 0.493 (p<0.001) was observed between awareness and practice scores.

**Conclusion:**

Seven and four out of 10 study participants had good awareness and practice scores about foot care, respectively. A tailor-made health education module addressing the lacunae identified in the awareness and practice domains needs to be provided to the patients with diabetes mellitus.

## Introduction

Diabetes mellitus (DM), a chronic disease of metabolism is estimated to have affected 463 million adults worldwide. Of these, one out of six adults are from India, accounting for around 77 million diabetic patients [1]. Untreated DM may lead to macro and microvascular complications including diabetic nephropathy, retinopathy, and peripheral neuropathy [2]. Ulcer of foot is one of the frequently observed complications among individuals with DM which often remains undetected and its incidence ranges from 8-17% [3]. Diabetic foot ulcers (DFU) are estimated to be responsible for around 85% of diabetes-related amputations. Micro-angiopathy, neuropathy, foot deformities and mechanical stresses are identified as the risk factors for the development of DFU. Furthermore, individuals with DFU are evidenced to experience reduced health-related quality of life [4].

Self-care practices include modifying diet, increasing physical activity, adherence to medication, monitoring of glucose levels in blood and care of the feet [5]. The activities pertaining to self-care performed by the patients are found to be associated with the outcomes of DM and development of complications. Individuals with DM are at constant risk of developing DFU. Appropriate measures including care from multidisciplinary diabetes care team as well as patient education helps in prevention of around 49-85% of diabetic foot related complications, thereby improving quality of life [6].

Tailor-made health education messages in the socio-cultural context of the patients may be useful to address the shortcomings in knowledge [7]. However, this requires assessment of the knowledge practice gaps in patients with DM. Hence, the aim of this study was to assess the awareness and practice regarding foot care and its associated factors among patients admitted with DM in a medical college hospital of coastal Karnataka, India.

## Methodology

### Study design and the participants

This cross-sectional study was conducted at Yenepoya medical college hospital, Mangaluru situated in coastal Karnataka, India from 24th December 2016 to 21st January 2017. The tertiary care hospital has 900 inpatient beds. Majority of the patients attending the hospital belong to the southern and central parts of Karnataka and northern Kerala.

### Inclusion criteria

All the adults aged >18 years with DM admitted for various complications in medical and surgical specialty wards and provided informed consent for voluntary participation in the study were included.

### Exclusion criteria

Seriously ill individuals (patients having cardiac complications like coronary artery disease/neurological complications like stroke) and pregnant women were excluded.

### Data collection

Data was obtained by interviewing the patients admitted in medical and surgical wards using a structured questionnaire by four trained medical interns under supervision.

### Questionnaire design and validation

We have adopted a structured and validated questionnaire which was used in a similar published study from Kerala [8]. The questions were translated to Kannada and a back translation to English was done. Content validity was performed by involving three subject experts proficient in both English and Kannada. Mean Content validity index (CVI) of the questionnaire was 0.927 and Cronbach’s α coefficient for reliability was 0.808. The questionnaire comprised of 4 sections i.e. socio-demographic variables, clinical variables, awareness and practice questions regarding foot care. There were 10 questions on awareness and 8 questions on practice assessment. Favorable response was scored ‘1’ and unfavorable response was scored ‘0’. Two questions under the section of practice assessment were given scores from 0 to 2. The maximum attainable scores for awareness and practice were 10 each. The scores 0-3 indicated poor score whereas ≥4 indicated good score for both awareness and practice. This categorization was based on a prior study from Indian context [8].

### Sample size calculation

Based on the finding that 75% of the patients with DM had a good knowledge score about foot care in Vellore, south India, [4] sample size of 317 was arrived with 5% alpha error, precision of 5% and expecting a 10% non-response rate. Non-probability purposive sampling method was employed in the selection of study participants.

### Outcome variables

Awareness and practice scores of patients regarding care of feet.

### Explanatory variables

Socio-demographic variables and clinical variables.

### Ethics approval

Approval of institutional ethics committee was obtained for the study (protocol no: 2016/372). Informed consent of written nature was obtained from the study participants after explaining the aim and nature of the study in their native language (Kannada/Malayalam). The research adhered to the principles of ethics in medical research involving humans as per the Helsinki declaration.

### Data management and statistical analysis

Data was entered in Microsoft excel and analysis was performed using Statistical Package for Social Sciences (SPSS Inc, Chicago, USA; Version 22.0). Descriptive statistics (frequencies, means and standard deviations, median and IQR) were used for demographic/clinical variables and to report participants’ awareness/practice scores. Bivariate logistic regression was used to determine the association between categorical demographic/clinical factors and awareness/practice scores. Linear regression was used to determine the association between continuous variables and awareness/practice scores. Pearson correlation was used to examine the correlation between awareness and practice scores. The p-value for significance was fixed as less than 0.05.

## Results

### Study characteristics

The study participants were aged 56.98 (±10.54) years and males constituted to 63.4%. Majority of the participants (88%) were married and most of them practiced Islam (46.7%) and Hinduism (43.8%). Half of the participants received education up to primary school and homemakers constituted 21.8% followed by semiskilled and unskilled workers (18% each). Majority of the participants belonged to below poverty line category (79.2%; Table 1).

Median duration of DM was 6 (4-10) years. The mean glycosylated hemoglobin level (HbA1C) was 8.09% (±2.07) and the participants were more likely to receive oral hypoglycemic medications (63.4%). Most of the subjects were taking medications regularly (84.5%), irregular medication intake (compliance <90% in the last month) was seen among 14.2% and 1.3% were defaulters (not taken medication in the last month). The most frequent complication was diabetic foot ulcer (34.4%) followed by coronary artery disease (7.9%) and peripheral neuropathy (7.3%).

### Awareness and practice regarding care of feet among patients with DM

In this study, 71.3% of the study participants believed that any infection in the feet could develop into a wound and 27.8% knew that patients with diabetes can develop gangrene of the feet. About 85% patients practiced washing their feet daily whereas 23% patients practiced changing their footwear timely. Other awareness and practice parameters assessed are presented in table 2.

The awareness and practice scores were computed by adding the scores. The median awareness score was found to be 5 (3-7). The median practice score was 3 (2-5). It was observed that 219/317 (69.1%) of the study participants had good awareness and 132/317 (41.6%) had good practices towards foot care. (Table 3) There were a few participants (8.2%) who had absolutely no awareness with score of zero and exhibited poor foot care practices (4.4%) where the practice score was zero.

### Associations between socio-demographic/clinical characteristics and awareness and practice scores

Good awareness scores regarding care of feet among the patients was associated with male gender (p=0.027), currently not married (p=0.044), socioeconomic status of below poverty line (p=0.014) and presence of ulcer of foot as a complication (p=0.021). Good practice scores was associated with secondary schooling and above (p=0.003) and patients currently receiving insulin with/without oral hypoglycemic agents (p=0.045). Among the continuous variables, awareness score decreased by 0.136 times with every one unit rise in HbA1C levels (p=0.042). (Table 3) There was a moderate correlation between awareness and practice scores with Pearson value of 0.493 (p-value < 0.001).

## Discussion

This study looked into the awareness and practice components related to care of feet among patients with DM admitted to medical college hospital using a previously validated scale. Foot self management is one of the cornerstones for preventing DFU. Patient education plays an important role in imparting knowledge and skills about foot care and it is predicted to reduce the DFU cases by 50% [6,9]. Even the patient empowerment theory places patients as focal points and make them the primary decision makers about self-care [10]. Assessment of the existing awareness and practices towards foot care among the patients will help physicians devise an appropriate health education intervention and this was one of the outcomes planned from this study.

### Awareness about foot care and associations

Seven out of every 10 individuals exhibited good awareness about foot care in our study. Some parameters like awareness regarding infection causing foot ulcers, sensation being lost and decreased blood flow causing ulcers had higher frequencies whereas patients scored less in the awareness of gangrene as a complication of DFU. Similar findings were recorded in studies from other developing countries [11,12]. One of the studies conducted at diabetic clinics in India showed a higher percentage of patients having good awareness scores which was attributed to good community diabetes care programme [4].

Male gender, currently not married, below poverty line status and presence of foot ulcer as a complication were associated with good awareness scores in our study. Poorly controlled DM, as evidenced by HbA1C was associated with poor scores. Female gender was seen as a better predictor of awareness in Bangladesh [11]. Higher educational levels were associated with better knowledge scores in other studies from the developing world [11-13]. Marital status was also observed to be associated in a study from Saudi Arabia [12]. Factors such as male sex, educational status of lower order and shorter duration of DM were associated with poor knowledge in a study from south India [4]. Another study from south India also reported that patients with DFU had poor knowledge scores as compared to those with no DFU [8]. We interpret that patients with DM who are females, married and those above poverty line need a special focus during delivery of health education about care of feet.

### Practice of care of feet and associations

Four out of every 10 individuals exhibited good practice of care of feet. This shows that there is a huge scope for improvement in this domain. Washing feet daily was the only practice which was observed to a great extent by the patients with DM. This may be because of the culturally practiced and accepted habit of washing the feet regularly by Indians. Some practices like trimming the nails straight and seeking health care in case of any abnormality over the feet were moderately observed. Other practices assessed were poorly followed. Suboptimal practices were also reported from other studies [13–17]. A study from southern part of India reported two thirds of the patients with a good practice score [4].

It is to be noted here that patients educated with secondary level and above were having good practice scores like many other studies [11,18,19]. Patients who were on insulin exhibited better scores which implies that they do have controlled DM and hence, the overall knowledge they possess has translated to better self-management. Longer duration of DM was a predictor of good practice in few published studies [11,12]. Awareness and practice scores were positively correlated in our study which was also reported from other studies [16,20].

Health education interventions result in improvement of knowledge and thereby attitudes. These changes will culminate in better preventive self-care practices [10]. Findings from a study revealed that one fourth patients with DM seldom paid attention to foot care in spite of having foot complications or major risk factors. Regular checking of the feet amongst these was related to receipt of education and examination of the feet [21]. Physicians, medical interns and nurses have a vital role to play in educating patients with chronic diseases about self-management in the hospitals. But, it is quite often overlooked especially in academic hospitals due to paucity of time and patient overload. In such a scenario, experiences from our institution suggest that group health education may be incorporated by deputing interns and trained para-medical staff. The health education may also be tried in peripheral health centers with medical officers, nurses and other paramedical staff as recommended by the national programme for non-communicable diseases in India [22]. Self learning module is another alternative which has proved effective in improving awareness and practices towards care of the feet [23].

## Limitations of the study

The hospital nature of the study makes the findings not generalizable. We also did not look into the attitudes as we felt that measuring this would be difficult especially among hospitalized patients with one or the other complication. Also, we did not study physician related factors with respect to poor foot care practices which has a scope for further research.

## Conclusion

Seven and four out of 10 study participants had good awareness and practice scores about foot care respectively. Good awareness scores were associated with male gender, currently not married, below poverty line status and presence of diabetic foot ulcer. Good practice scores were associated with patients with secondary education and above and those receiving insulin. There was a moderate correlation between awareness and practice scores. Patient education needs to consider these factors when it is delivered.

### Future scope of the study:

The effectiveness of health education provided to the diabetics using a structured module which is culturally acceptable needs to be evaluated both quantitatively and qualitatively. Regular follow-ups and complication prevention can be facilitated by scheduled reminders to the patients and this is an area of future research.

### What is already known on this topic?

Deficiencies in awareness and practice components of care of the feet among patients with DM are established by multiple studies conducted at hospitals and diabetic clinics. Some studies have looked into the factors influencing them in various contexts.

### What this study adds?

The lacunae in the awareness and practice domains were elicited by compiling the responses obtained to the questions. Hence, these aspects should be focused upon while developing the health education module. Factors associated with good/poor awareness and practice scores were obtained. There is a positive correlation between awareness of diabetes foot care being translated to good foot care practice.

## Figures and Tables

**Table 1:** Socio-demographic and clinical characteristics of patients with diabetes mellitus who were assessed for awareness and practice regarding care of feet at Yenepoya medical college hospital, Karnataka, India (n=317)

Variables	Frequency	Percentage
**Gender**		
Male	201	63.4 (62.1-64.7)
Female	116	36.6 (35.3 – 37.9)
**Religion**		
Hinduism	139	43.8 (42.42 – 45.18)
Islam	148	46.7 (45.51 – 48.09)
Christianity	24	7.6 (7.2 – 7.9)
Others	6	1.9 (1.7 – 2.0)
**Marital status**		
Not married	**6**	**1.9 (1.7 – 2.0)**
Married	279	88.0 (87.41 – 88.59)
Widow/widower	24	7.6 (7.2 – 7.9)
Divorced	8	2.5 (2.37 – 2.63)
**Educational status**		
Not literate	74	23.3 (22.3 – 24.3)
Up to primary school	162	51.1 (49.7 – 52.5)
Secondary school	60	19.0 (18.14 – 19.86)
Pre university/diploma and above	21	6.6 (6.26 – 6.94)
**Occupational status**		
Semi-professional	16	5.0 (4.74 – 5.26)
Skilled	50	15.8 (15.06 – 16.54)
Semi-skilled	57	18 (17.17 – 18.83)
Unskilled	57	18 (17.17 – 18.83)
Homemaker	69	21.8 (20.85 – 22.75)
Retired	16	5.0 (4.74 – 6.26)
Unemployed	52	16.4 (15.63 – 17.17)
**Socio-economic status**		
Above poverty line	66	20.8 (19.87 – 21.72)
Below poverty line	251	79.2 (78.27 – 80.12)
**Current treatment**		
Oral hypoglycemic drugs	201	63.4 (62.1 – 64.7)
Insulin	65	20.5 (19.59 – 21.41)
Both	51	16.1 (15.35 – 16.85)
**Medication regularity in the last one month**		
Regular	268	84.5 (83.77 – 85.23)
Irregular	45	14.2 (13.52 – 14.88)
Defaulter	4	1.3 (1.23 – 1.37)

**Table 2:** Awareness and practice regarding care of feet among the patients with diabetes mellitus at Yenepoya medical college hospital, Karnataka, India (n=317)

Awareness-related foot care	Number	Percentage
Patients with DM will have decreased flow of blood in their feet	127	40.1 (38.75 – 41.45)
Patients with DM will have lack of sensations in their feet	161	50.8 (49.4 – 52.2)
Patients with DM will have ulcers in their feet	158	49.8 (48.4 – 51.2)
Patients with DM develop gangrene	88	27.8 (26.68 – 28.92)
Receipt of information regarding foot care in the past	139	43.8 (42.42 – 45.18)
Smoking can decrease the blood flow to feet	135	42.6 (41.23 – 43.97)
Loss of sensation in feet makes them more prone to have ulcers	195	61.5 (60.17 – 62.83)
Reduced blood flow makes them more prone to get foot ulcers	195	61.5 (60.17 – 62.83)
Any infection in the feet may develop to wounds	226	71.3 (70.16 – 72.44)
Appropriate method of trimming toe nail	157	49.5 (48.1 – 50.9)
		
**Practice-related foot care**	**Number**	**Percentage**
Washing feet daily	269	84.9 (84.18 – 85.62)
Moisturizing dry areas of feet	87	27.4 (26.29 – 28.51)
Checking feet daily for any injury	98	30.9 (29.74 – 32.06)
Seeking health care if any abnormality on feet is noted	184	58 (56.64 – 59.36)
Cutting the toe nails cut straight through	163	51.4 (50 – 52.8)
Checking for shoes/socks leave marks on the feet	75	23.7 (22.69 – 24.71)
Practice of changing footwear once/more than once in a year	73	23 (22.01-23.99)
Practice of going for foot check up	88	27.8 (26.68 – 28.92)

**Table 3:** Association between socio-demographic/clinical characteristics and awareness and practice regarding care of feet among patients with diabetes mellitus at Yenepoya medical college hospital, Karnataka, India (n=317)

Categorical variables		Awareness						Practice					
		Good	Poor	Crude OR (95% CI)	p-value	aOR (95% CI)	p-value	Good	Poor	Crude OR (95% CI)	p-value	aOR (95% CI)	p-value
**Gender**	Male	150 (74.6)	51 (25.4)	2.003 (1.230-3.264)	**0.005**	2.105 (1.089-4.068)	**0.027**	89 (44.3)	112 (55.7)	1.349 (0.844-2.155)	0.210	0.894 (0.482-1.661)	0.724
	Female	69 (59.5)	47 (40.5)	1		1		43 (37.1)	73 (62.9)	1		1	
**Marital status**	Married	189 (67.7)	90 (32.3)	0.560 (0.247-1.271)	0.161	0.404 (0.167-0.976)	**0.044**	116 (41.6)	163 (58.4)	0.979 (0.492-1.944)	0.951	0.837 (0.397-1.763)	0.639
	Currently not married	30 (78.9)	8 (21.1)	1		1		16 (42.1)	22 (57.9)	1		1	
**Educational status**	Secondary school and above	62 (76.5)	19 (23.5)	1.642 (0.919-2.935)	0.092	1.664 (0.890-3.109)	0.110	46 (56.8)	35 (43.2)	2.292 (1.372-3.830)	**0.001**	2.249 (1.312-3.857)	**0.003**
	Up to primary school	157 (66.5)	79 (33.5)	1		1		86 (36.4)	150 (63.6)	1		1	
**Occupational status**	Currently employed	128 (71.1)	52 (28.9)	1.244 (0.771-2.009)	0.371	1.026 (0.533-1.975)	0.938	80 (44.4)	100 (55.6)	1.308 (0.831-2.058)	0.246	1.356 (0.746-2.467)	0.318
	Currently not employed	91 (66.4)	46 (33.6)	1		1		52(38)	85(62)	1		1	
**Socio-economic**	Above poverty line	38 (57.6)	28 (42.4)	0.525 (0.3-0.920)	**0.023**	0.472 (0.259-0.862)	**0.014**	30 (45.5)	36 (54.5)	1.217 (0.705-2.102)	0.480	1.082 (0.608-1.926)	0.788
**status**	Below poverty line	181 (72.1)	70 (27.9)	1		1		102 (40.6)	149 (59.4)	1		1	
**Complication**	Foot ulcer	86 (78.9)	23 (21.1)	2.109 (1.229-3.619)	**0.006**	1.966 (1.109-3.595)	**0.021**	51 (46.8)	58 (53.2)	1.379 (0.863-2.202)	0.178	1.343 (0.801-2.250)	0.263
	Other than foot ulcer	133 (63.9)	75 (36.1)	1		1		81 (38.9)	127 (61.1)	1		1	
**Current treatment**	Insulin with/without oral hypoglycemic agents	81 (69.8)	35 (30.2)	1.057 (0.643-1.735)	0.828	0.832 (0.486-1.426)	0.504	57 (49.1)	59 (50.9)	1.623 (1.022-2.578)	**0.040**	1.654 (1.010-2.708)	0.045
	Only oral hypoglycemic agents	138 (68.7)	63 (31.3)	1		1		75 (37.3)	126 (62.7)	1		1	
**Medication regularity**	Irregular	35 (71.4)	14 (28.6)	1.141 (0.583-2.233)	0.699	1.204 (0.597-2.424)	0.604	15 (30.6)	34 (69.4)	0.569 (0.296-1.095)	0.089	0.557 (0.285-1.088)	0.087
	Regular	184 (68.7)	84 (31.3)	1		1		117 (43.7)	151 (56.3)	1		1	
**Continuous variables**		**Awareness**						**Practice**					
		**Coefficient** **(95% CI)**				**p-value**		**Coefficient** **(95% CI)**				**p-value**	
**Age**		-0.082 (-0.063-0.016)				0.245		0.035 (-0.02-0.034)				0.622	
**Years lived with diabetes**		0.077 (-0.029-0.1)				0.278		0.0 (-0.044-0.044)				0.999			
**Glycosylated hemoglobin levels**		-0.136 (-0.408-(-0.008))				**0.042**		-0.070 (-0.209-0.064)				0.298	

OR=Odds Ratio, aOR=Adjusted Odds Ratio, CI=Confidence Interval
